# Atypical brain response to novelty in rural African children with a history of severe falciparum malaria

**DOI:** 10.1016/j.jns.2010.05.018

**Published:** 2010-09-15

**Authors:** Michael Kihara, Michelle de Haan, Harrun H. Garrashi, Brian G.R. Neville, Charles R.J.C. Newton

**Affiliations:** a The Centre for Geographical Medicine Research (Coast), Kenya Medical Research Institute, Kilifi, Kenya; b Developmental Cognitive Neuroscience Unit, Institute of Child Health, University College London, UK; c Neurosciences Unit, The Wolfson Centre, Institute of Child Health, University College London, UK; d Clinical Research Unit, London School of Hygiene and Tropical Medicine (LSHTM), London, UK

**Keywords:** Severe falciparum malaria, Event-related potentials, Cognitive, Children

## Abstract

*Plasmodium falciparum* is the most common parasitic infection of the central nervous system causing neuro-cognitive deficits in 5–26% of paediatric cases. The burden cannot be reliably estimated because of lack of sensitive, culture-fair and robust assessments in rural settings. Auditory and visual brain event related potentials (ERPs) are used to compare novelty processing in children exposed to severe malaria with community controls. Fifty children previously admitted and discharged from Kilifi District Hospital with severe falciparum malaria were selected and compared with 77 unexposed agematched children. The results showed that up to 14% of children exposed to severe malaria had significantly different responses to novelty compared to unexposed children. Children exposed to severe malaria had smaller P3a amplitudes to novelty in both auditory [*F* (3, 119) = 4.545, *p* = 0.005] and visual [*F* (3, 119) = 6.708, *p* < 0.001] paradigms compared to unexposed children. In the auditory domain the differences in processing of novelty were not related to early component processing. The percentage of children with severe malaria showing impaired performance using ERPs is within the range previously reported using neuropsychological tests. The overall pattern suggests that severe malaria affects prefrontal and temporal cortices normally activated by stimulus novelty.

## 1 Introduction

Severe falciparum malaria is caused by *Plasmodium falciparum*, which is the most common parasitic infection of the central nervous system [1] and is known to cause cognitive impairment in children [2]. Seizures, prostration and impaired consciousness are common symptoms of severe malaria and have been associated with poor cognitive outcome [3–5]. Cerebral malaria (CM) is the most severe neurological presentation of falciparum malaria and it is characterised by coma [6]. Several retrospective studies in Africa have shown that between 5 and 26% of children with a history of severe falciparum malaria will have persistent cognitive deficits [3–5,7–12].

Most of the cognitive evaluation of children in sub-Saharan Africa (SSA) has relied on the use of adaptations of Western neuropsychological tests. Several studies have used such tools to examine how CM, affects cognitive development. These studies have demonstrated deficits in areas of memory [3,7,10,11], attention [3,5,11], executive functions [10,11] and visuo-spatial skills [3,9,11]. However, the high rate of illiteracy amongst poor rural populations, language barriers and the fact that children rarely interact with adult strangers do complicate the use of neuropsychological tests particularly in rural areas [13]. Furthermore, the use of adapted neuropsychological tests, rather than tests developed for local populations is thought to introduce bias in the reporting and interpretation of these tests [2,4]. Despite these limitations, neuropsychological tests are useful in determining functions that may be impaired by exposure to damaging infections in our setting. However, assessing multiple levels of function by including neuroimaging or neurophysiological assessments may help to provide a more complete picture of the neuro-cognitive consequences of severe falciparum malaria.

Relatively little is known about the mechanisms and neural correlates of cognitive outcomes following severe malaria [14]. The few existing studies suggest that both diffuse cerebral atrophy and focal cortical atrophy can be observed months to years following the acute event, with focal atrophy typically consistent with focal seizures during the acute event [15,16]. To date such reports are based on relatively small numbers of cases (15 or less) and without a detailed examination of how brain structural abnormalities relate to cognitive outcome.

Event-related brain potentials (ERPs) are a potentially useful approach to investigate neuro-cognitive outcome following paediatric severe malaria, because: (a) they can provide measures of neuro-cognitive function that are less influenced by motor or language skills, and (b) compared to alternatives such as functional magnetic resonance imaging, they are relatively easy-to-obtain and cost-effective measures of neural activity related to cognitive function. ERPs are voltage fluctuations that are associated in time with a physical (visual, audio or smell) or mental occurrence (attention, recognition, memory, etc; [17]. These brain potentials can provide very precise information about the timing of brain activation and some information about the spatial location of the regions activated. Thus, ERPs can potentially provide more direct insight than neuropsychological measures into the stage of information-processing affected by cerebral disease and some ideas about the brain regions affected. ERPs have frequently been used to assess the development of sensory and cognitive processing in Western children during normal development and disease or disorder [18,19].

The brain's response to novel or unexpected stimuli has been commonly studied in infants and children as it is a reliable and robust response and because orienting to novel information in the environment is considered a fundamental process in cognitive development. Studies with adults suggest that a fronto-temporal brain network is involved in processing novel events. Recently we used ERPs to evaluate auditory and visual processing of novelty in 178 normally developing children aged 4–12 years living in rural Kenya [20]. We found that typical ERP components observed in studies of European and American children were also elicited in rural Kenyan children, with evidence of novelty detection in both modalities and of age-related changes in the amplitudes and latencies of sensory and cognitive components. In the current study, we used the same paradigms in children exposed to severe falciparum malaria in rural Kenya to determine whether ERPs would reveal impairments and to gain insight into the nature of atypical brain processing following severe malaria.

## 2 Methods

The study took place between August 2004 and March 2005 in Kilifi District Hospital (KDH) located on the Kenyan coast. KDH is the main government district hospital and caters for about 80% of the local population. Sixty-four children, aged between 6 and 7 years who were admitted to KDH between May 2002 and March 2004 with severe falciparum malaria were selected from the hospital database. The inclusion criteria for the diagnostic groups were:

1. Admission to hospital with cerebral malaria, CM (defined as Blantyre Coma Score (BCS) of ≤ 2 for 4 or more hours [21], a peripheral falciparum malaria parasitaemia and exclusion of other causes of encephalopathy) [22].
2. Children who had been admitted with a primary diagnosis of malaria with seizures, MS (defined as a peripheral falciparum malaria parasitaemia with > 2 seizures within 24 h or focal or prolonged > 30 min) who did not lapse into coma.
3. Children admitted having malaria with prostration, PM (defined as peripheral parasitaemia, a BCS of 3 or 4 and child is unable to sit or walk without support).

Fourteen children were subsequently excluded due to either co-infections/misdiagnosis (6 had acute bacterial meningitis, 4 had febrile seizures and 3 with viral encephalitis excluded on the basis of a CSF cell count of more than 20 cells/mm^3^) or excessive EEG artefact (1 child). The 50 children (mean = 6 years 7 months, SD = 6 months, males = 23) with severe falciparum malaria [CM = 27, MS = 14 and PM = 9] were compared with 77 community controls, males = 38) that did not have a history of being admitted with severe malaria and had no history of neurological impairment detected by a screening questionnaire [23]. These controls (CT) were part of a normative study on novelty detection of normally-developing children in the same community [20]. Each child was screened for hearing and vision using Kamplex audiometer (PC Werth, UK) and Sonksen–Silver chart [24] respectively, before recording auditory and visual ERPs and all judged to have abilities within normal limits.

The children we studied had different complications of severe falciparum malaria but were similar on most background characteristics (Table 1).

The study was approved by the National Ethics Committee of Kenya and parents gave consent for study staff to record ERPs of their children. The ERP technicians (MK and HH) were blinded to the group status of the children.

## 3 Auditory paradigm

We used a non-response 3-stimulus ‘novelty oddball’ paradigm consisting of two types of repeated sounds, frequent and infrequent tones, and novel sounds. These tones and novel sounds were presented through two speakers placed in front of the children next to the monitor using *Presentation®* software (Neurobehavioral Systems, USA). Ten percent of the stimuli were infrequent tones (2000 Hz, 200 ms long, 5 ms rise and fall time, 70 dB Sound Pressure level, SPL), 10% were composed of environmental noises e.g. dog bark, bell ring etc and the remainder were frequent tones (1500 Hz, 200 ms long, 5 ms rise and fall time, 70 dB SPL). The frequent stimuli immediately prior to each infrequent stimulus were selected for averaging to provide similar signal-to-noise ratios. Novel sounds were digitally adjusted in intensity so that they did not exceed 70 dB SPL as determined using a Bruel and Kjaer sound pressure meter. Two-blocks of 700 stimuli each were presented with a short break in between. The children were not given any instructions other than fix their gaze on the computer monitor. A cartoon picture appeared at the centre of the computer monitor to attract the children's gaze and avoid unnecessary head movements. The duration of the tones/noises was 200 ms with a rise and fall time of 5 ms and a stimulus onset asynchrony (SOA) of 700 ms.

## 4 Visual paradigm

The visual experiment was a non-response 3-stimulus paradigm consisting of 2 female faces posing neutral expressions and non-face novel stimuli of abstract paintings (Kandinski's art). All stimuli were in colour photographic slides and were of equal size and presented at a visual angle of 16.8 × 14.3°. Two blocks of 100 trials were presented in a random order, with 60% of the trials showing the frequent face, 20% infrequent face, and 20% novel stimuli. Novel stimuli were trial unique. Subjects were not given any instructions other than look at the computer screen. The duration of the image presentation was 200 ms with an inter-stimulus interval of 3000 ms.

## 5 Data acquisition and analysis

Each child sat on an easy chair in a partially lit, sound-attenuated room looking towards a computer monitor placed 70 cm away with two loud-speakers beside it. Data was recorded from midline leads (Fz, FCz, Cz, Pz), posterior temporal electrodes (T5, T6) as well as mastoid processes (A1, A2) using the standard 10–20 electrode placement system [25]. Horizontal and vertical electro-oculographs were monitored by two electrodes on the outer canthus of the eye and just below the eye respectively. All locations were referenced to a common Cz reference and grounded at Fpz, and later re-referenced to averaged mastoids reference for purposes of comparison with previous studies employing this reference. All impedances were less or equal to 8.2 kΩ. Electroencephalographic (EEG) and EOG channels were recorded using Neuroscan® version 4.3 acquisition system and amplified with Nu-amps (Neuroscan Labs, El Paso, USA). Continuous EEG data were recorded at a sampling rate of 500 Hz with a bandpass of 0.1 to 70 Hz and low pass filtered offline at 20 Hz. The continuous file was screened for muscular and ocular artifacts and then segmented into epochs from −200 to 1000 ms and −200 to 1500 for auditory and visual stimuli respectively. An ocular artefact reduction algorithm on the Scan 4.3 software (Neuroscan Labs) was used to remove artefacts due to blinking. Any trials with amplitude deflections exceeding ± 100 μV were rejected. A minimum of 20 trials for each stimulus was required for inclusion of an individual average ERP waveform. The EEG data of one child was excluded due to excessive artefact. All data was analysed using SPSS for Windows, version 15 (SPSS Inc®, Chicago, USA). Within-subject factors included Site (Fz, FCz, Cz and Pz) and stimuli (frequent, infrequent and novel) for all components except the visual N170 where the variable Site had the levels T5, T6. The between-subject factors were Diagnosis (CM, MS, PM or controls, CT) and Sex (male or female). Initial analyses showed no main effects or interactions with Sex therefore this variable will not be discussed further. The Greenhouse–Geisser correction is reported where applicable. We used the Tukey–Kramer test in the *post-hoc* analyses to correct for unequal sample sizes. Impairment was defined as 2 standard deviations from the mean of unexposed children and the level of significance was set at *p* < 0.05.

Time windows for ERP components were identified by guidance from our prior study [20] and by inspection of the grand-averages and individual subjects' averages to ensure that relevant peaks fell within the time window. In the auditory paradigm, the P1, N2 and P3a components were analysed. The P1 component was defined as the highest peak between 60 and 130 ms post stimulus presentation and the N2 component was defined as the most negative point between 120 and 220 ms. The P3a component was defined as the most positive point occurring between 250 and 450 ms. In the visual paradigm, we recorded the P3a, Nc and the face-sensitive N170 component. The P3a was the highest peak between 270 and 450 ms and Nc was the average amplitude between 300 and 850 ms. The N170 was defined as the most negative peak between 170–250 ms and was measured at occipito-temporal sites T5 and T6.

## 6 Results

### 6.1 Response to novelty

#### 6.1.1 Visual

**Fig. 1:**
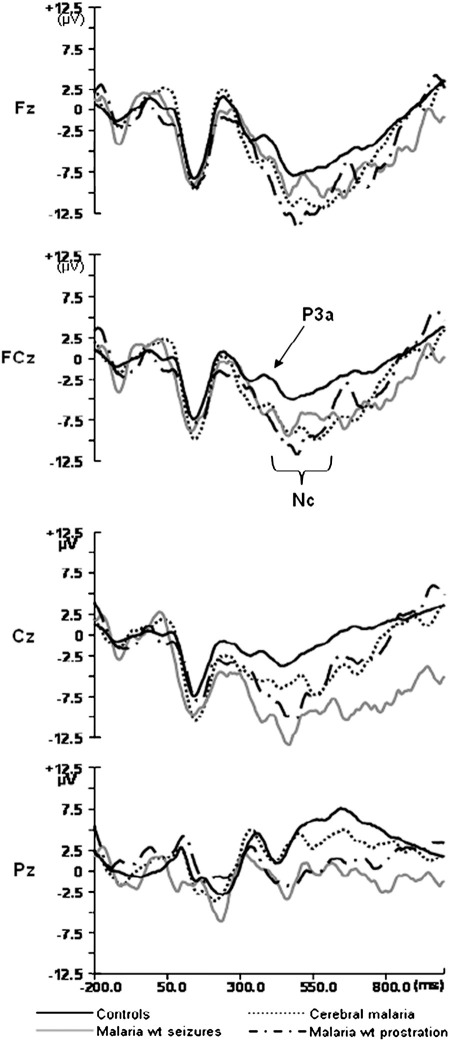
Grand averaged ERPs elicited by novel visual stimuli for each diagnostic group at each midline electrode.

To examine whether the response to novel pictures differed among the groups, we computed ANOVAs with between-subject factor of Diagnosis (CM, MS, PM, CT) and within-subjects factor of Site (Fz, FCz, Cz, Pz) for the amplitude and latency of the P3a and the mean amplitude of the Nc to novel pictures. Fig. 1 shows comparisons of the diagnostic groups to the novel pictures.

The amplitude of the visual P3a had a main effect of Diagnosis [*F* (3, 122) = 3.766, *p* = 0.013], with *post-hoc* analysis revealing that children exposed to severe malaria had smaller P3a amplitudes compared to controls (MS < CT, *p* = 0.004; PM < CT, *p* = 0.034). There was an interaction of Diagnosis by Site for amplitude of the Nc [*F* (9, 363) = 2.368, *p* = 0.040]. The interaction occurred due to larger Nc amplitudes at Fz than other electrodes (FCz, Cz and Pz) in controls and children with CM and PM, but not in children with exposure to MS. The latency of the visual P3a was not affected by exposure to severe malaria (*p* = 0.863).

We also examined how many children within each group showed atypical processing of visual novelty, by defining atypical processing as a response greater than 2 standard deviations from the control group mean (see Table 2). Eleven percent of children with CM had increased P3a latencies and 7% reduced P3a amplitudes compared to unexposed children, with none of the children in the PM or MS groups falling in this range and none of the groups doing so for Nc amplitude.

#### 6.1.2 Auditory

**Fig. 2:**
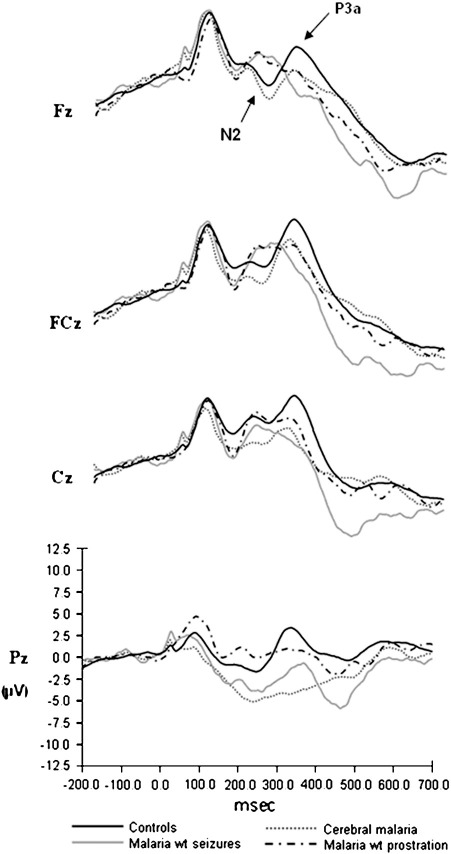
Grand averaged ERPs elicited by novel auditory stimuli for each diagnostic group at each midline electrode.

To examine whether the response to novel sounds differed among the diagnostic groups, we computed ANOVAs with between-subject factor of Diagnosis (CM, MS, PM or CT) and within-subjects factor of Site (Fz, FCz, Cz, Pz) for the amplitude and latency of the N2 and P3a to novel sounds. Fig. 2 shows comparisons of the diagnostic groups to the novel stimuli.

The N2 amplitude did not reveal any diagnostic effects (*p* = 0.365) but there was a significant main effect of Diagnosis on the P3a amplitude [*F* (3, 123) = 5.536, *p* = 0.001]. *Post-hoc* analysis revealed significantly smaller amplitudes for children with exposure to CM and MS compared to controls (*p* = 0.008 and *p* = 0.023 respectively) but not for children exposed to PM (*p* = 0.947) (Fig. 2).

There was no effect of Diagnosis on either the N2 latency (*p* = 0.139) or the P3a latency (*p* = 0.212).

We also examined how many children within each group showed atypical processing of auditory novelty, by defining atypical processing as a response greater than 2 standard deviations from the control group mean (see Table 3). This showed that 14% of children with a history of MS, 11% of those exposed to PM and 4% of those exposed to CM had atypically small P3a components.

### 6.2 Early-latency processing

To examine whether the altered processing of novelty observed in the groups with severe malaria may be due to differences in perceptual processing proceeding the auditory N2/P3a and visual P3a/Nc components, ANOVAs with between-subject factor of Diagnosis (CT, CM, MS, PM) and within-subjects factor of electrode (Fz, FCz, Cz, Pz for P1 and T5, T6 for N170) were conducted for the amplitude and latency of early-latency auditory (P1) and visual (N170) components.

#### 6.2.1 Visual N170

There was an electrode by diagnosis interaction for the N170 amplitude [*F* (3, 118) = 3.182, *p* = 0.027]. This interaction was due to a significantly smaller N170 amplitude at T5 for children exposed to MS compared to controls (*p* = 0.014) but the same was not true for those exposed to CM or PM (*p* = 0.712 and *p* = 0.067 respectively). There was no significant effect of Diagnosis on the latency of the N170 component (*p* = 0.702).

#### 6.2.2 Auditory P1

There was no significant main effect or interaction involving Diagnosis for either the amplitude or the latency of the P1 component [*p* = 0.892 and 0.444 respectively] (Fig. 2).

### 6.3 Response to novel compared to familiar stimuli

To determine whether the altered processing of novel stimuli in children with malaria was associated with a failure to detect novelty, we computed ANOVAs with within-subjects factors of Stimulus (Novel, Frequent, and Infrequent) and Site (Fz, FCz, Cz, Pz) for the amplitude and latency measures of the auditory N2/P3a and visual P3a/Nc separately for each group. We present results from the control group first to establish the normal pattern, and then each of the three groups of severe malaria.

#### 6.3.1 Control group

**Fig. 3:**
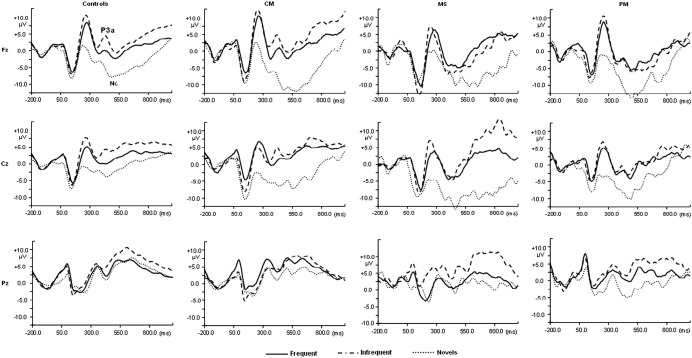
Grand averaged waveforms for frequent, infrequent and novel visual stimuli at midline electrodes for diagnostic groups. CM represents children with cerebral malaria, MS are children with malaria with seizures and PM had malaria with prostration.

In the visual paradigm, both the P3a amplitude and latency had significant Stimulus effects [*F* (2, 152) = 20.817, *p* < 0.001 and *F* (2, 152) = 5.745, *p* = 0.004 respectively]. P3a amplitudes were larger for infrequent stimuli compared to novel stimuli (Table 4) while latencies where shorter for novel stimuli compared to either frequent or infrequent stimuli (Table 4). The Nc also had a significant main effect of Stimulus [*F* (2, 152) = 31.243, *p* < 0.001] which occurred due to larger Nc amplitudes to novel stimuli compared to both frequent and infrequent stimuli (Fig. 3).

**Fig. 4:**
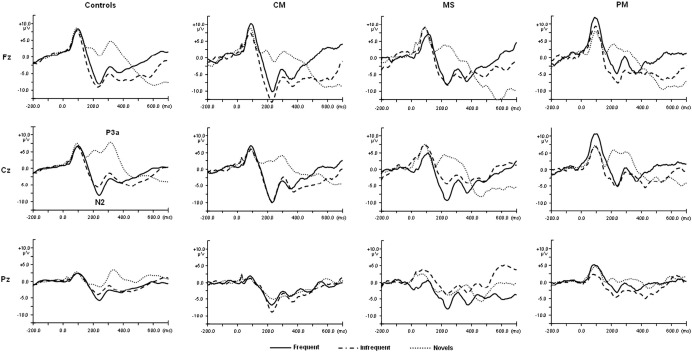
Grand averaged waveforms for frequent, infrequent and novel auditory stimuli at midline electrodes for diagnostic groups. CM represents children with cerebral malaria, MS are children with malaria with seizures and PM had malaria with prostration.

The control group revealed significant Stimulus effects for both the N2 amplitude [*F* (2, 152) = 74.290, *p* < 0.001] and P3a amplitude [*F* (2, 152) = 86.303, *p* < 0.001]. These occurred due to smaller N2 amplitudes and larger P3a amplitudes associated with novel stimuli compared to familiar stimuli (Table 5 and Fig. 4). The latency of the N2 component had a significant stimulus effect [*F* (2, 152) = 6.510, *p* = 0.003] due to shorter latencies associated with infrequent stimuli compared to novel stimuli (Table 5). There were no effects of Stimulus or Site for the P3a latency.

In summary, typically developing children showed smaller N2s and larger P3as to novel compared to frequent sounds and slower N2s for novel compared to infrequent sounds. They also showed larger and slower P3as to infrequent pictures and larger Ncs to novel pictures.

#### 6.3.2 CM group

In the visual paradigm, the P3a amplitude had a main effect of stimulus [*F* (2, 52) = 7.769, *p* = 0.002] but the latency did not reach significance (*p* = 0.148). P3as were smaller for novel compared to frequent stimuli. The Nc revealed a significant Stimulus effect [*F* (2, 52) = 13.269, *p* < 0.001] due to larger amplitudes for novel compared to both frequent and infrequent stimuli. In the auditory paradigm, the N2 amplitude revealed a significant effect of stimulus [*F* (2, 52) = 20.388, *p* < 0.001] but none for the N2 latency (*p* = 0.519). N2 amplitudes were smaller for novel compared to infrequent stimuli. The P3a amplitude also showed a significant effect of stimulus [*F* (2, 52) = 8.392, *p* = 0.002], but none for the P3a latency (*p* = 0.584) due to larger amplitudes for novel compared to familiar stimuli.

In summary, children exposed to CM failed to show stimulus effects on latency of the auditory N2 and visual P3a observed in the controls.

#### 6.3.3 MS group

The visual P3a did not reveal any significant main effect of Stimulus (*p* = 0.711), but there was a significant effect for the P3a latency [*F* (2, 26) = 4.614, *p* = 0.021] due to shorter latencies for novel compared to familiar stimuli. The Nc component revealed a main effect of stimulus [*F* (2, 26) = 3.937, *p* = 0.041] due to larger amplitudes associated with novel compared to either frequent or infrequent stimuli. In the auditory paradigm, the N2 amplitude had a significant main effect of Stimulus [*F* (2, 26) = 6.039, *p* = 0.016] but not the N2 latency (*p* = 0.442). N2 amplitudes were smaller for novel compared to infrequent stimuli. The P3a component did not reveal any Stimulus effects for either the amplitude (*p* = 0.169) or latency (*p* = 0.119).

In summary, children with MS did not show effects on auditory N2 latency, auditory P3a amplitude and visual P3a amplitude observed in controls.

#### 6.3.4 PM group

In the visual paradigm there were no Stimulus effects on the amplitude (*p* = 0.151) of the P3a but there were effects for latency (*F* (2, 16) = 4.506, *p* = 0.043) due to shorter latencies for the novel compared to the familiar stimuli. There was a significant main effect of Stimulus on the Nc component (*F* (2, 16) = 5.474, *p* = 0.024) due to larger amplitude for novel compared to familiar stimuli. In the auditory paradigm, children with exposure to PM also had a significant main effect of stimulus [*F* (2, 16) = 19.067, *p* < 0.001] on the N2 amplitude but not the N2 latency (*p* = 0.099). N2 amplitudes were smaller for novels compared to infrequent stimuli. The P3a component did not reveal any Stimulus effects in either the amplitude (*p* = 0.079) or the latency (*p* = 0.519).

In summary, children with PM did not show the effects for auditory N2 latency, auditory P3a amplitude, and visual P3a amplitude observed in controls.

Table 6 summarises the comparison of novel and familiar stimuli of children in the various diagnostic group and highlights the differences between exposed children and controls.

## 7 Discussion

The aim of the present study was to determine novelty processing and examine whether ERPs provide additional insights into the neuro-cognitive processing in Kenyan children exposed to severe falciparum malaria. We used auditory and visual novelty processing paradigms that we previously successfully applied to children in SSA and examined effects of diagnosis on attention orienting to novelty indexed by the auditory N2, P3a and visual P3a and Nc ERP components. We conducted further analyses to determine whether the observed differences in exposed compared to unexposed children were due to differences in early-latency sensory processing and whether they reflected an altered attention response to novelty or a failure to detect novelty. Overall the results provided evidence of neuro-cognitive impairment in up to 14% of children exposed to severe malaria, a figure within the range previously reported in neuropsychological studies of cognitive impairment following exposure to severe malaria in children [4,5,7,8,12]. The results of the present study suggest that children exposed to severe malaria show normal early auditory processing and initial detection of novelty but atypical further processing of auditory novelty as reflected in the P3a. These children also show atypical visual novelty processing, but these may to some extent be influenced by additional differences in early visual processing. Overall, this pattern is consistent with prior reports indicating that impairments in attention are common following exposure to severe malaria [7,12], and are suggestive of atypical processing in fronto-temporal brain networks involved in orienting attention to novelty.

### 7.1 Visual novelty processing

Our group analysis of the visual response to novelty showed that children with MS and PM, showed a reduced P3a to novelty compared to controls. The MS and PM groups also failed to show a differential P3a to novel compared to familiar pictures displayed by the control and CM groups, suggesting difficulties in initial orienting of attention to salient events. While children with CM did not, as a group, show impairments in P3a, at an individual level 11% showed atypical latencies and 4% atypical amplitudes, indicating that a subgroup of children with CM also showed difficulties in initial orienting of attention.

All groups showed evidence of novelty detection at longer latencies, with larger magnitude Ncs for novel pictures, with all groups except the MS group showing a stronger response to novelty frontally. The Nc is believed to be generated in frontal brain regions, a hypothesis supported by source analyses carried out on infant ERPs [26] and indirectly by parallels observed in the timing of developmental changes in Nc amplitude and the course of frontal cortical synaptogenesis [27,28]. The visual Nc is thought to represent attention alerting mechanisms in response to salient visual stimuli [29]. The results suggest that children with exposure to MS may activate a different, possibly broader and less frontally localised, brain network when attending to salient visual stimuli, though this conclusion remains speculative due to the low-density electrode array. We cannot completely rule out the possibility that these differences are influenced by earlier differences in sensory processing, as children with MS showed a reduced amplitude N170 over the left hemisphere with children with PM showing a similar trend (*p* = 0.06).

### 7.2 Auditory novelty

Our analysis of the N2-P3a complex showed both intact and atypical responses in exposed to unexposed children. The N2 to novelty showed no effects of Diagnosis and its amplitude was smallest for novel stimuli in all groups. N2 is influenced by deviation in form or context of a prevailing stimulus [30], and is thought to be generated by diverse brain areas including the frontal and parietal cortical fields [31], the superior temporal planes and Heschl's gyrus [32]. These results, together with the lack of effect of Diagnosis on the earlier-latency P1, suggest that the initial sensory processing of the sounds and detection of the novel stimulus was largely unaffected by malaria. However, later-latency processing of auditory novelty reflected in the P3a showed a different result. Children with CM and MS showed reduced amplitudes of the P3a to novelty. Children with MS and PM showed no differential P3a to novel compared to familiar sounds. The P3a component is thought to represent involuntary attention to salient or novel events [33,34] and represents an orienting response [35,36]. The P3a component is attenuated in patients with lesions of the dorsolateral prefrontal cortex [37–39], and the temporal lobe [40,41], suggesting that this component is likely to be generated by a neural network involving the temporal and frontal lobes. Our results suggest that children with CM and MS show a reduced activation of this network to novelty sounds while children with MS and PM fail to show differential activation of this network by novelty.

### 7.3 Limitations of the study

The results of the present study should be interpreted conservatively based on a number of limitations. The selection of children with cerebral malaria was made using the Blantyre coma score (Molyneux et al., 1989) and recent developments show that this could lead in misdiagnosis in up to 25% of the cases (Taylor et al., 2004). Future studies should aim to use malaria-specific retinopathies to clearly diagnose children with CM (Beare et al., 2006).

This study also raised some questions regarding differences in processing among the malaria sub-groups. Based on prior neuropsychological reports, it would be reasonable to expect that the CM group would show stronger and more consistent impairments but this was not always the case. For example, the PM and MS groups showed more impairment in differentiation of novel compared to familiar stimuli, and they also tended to show higher rates of impairment when this was assessed at the individual level. This may reflect a true pattern, as some previous studies have shown a similar pattern, such that children with malaria and complicated seizures (MS) have greater impairments on speech and language subtests, than children with a history of cerebral malaria (Carter et al., 2005). However, there are several other possible reasons for these unanticipated findings. First, they may be due to misdiagnosis due to absence of techniques like the positive malaria retinopathies. Second, they may have occurred because the neural networks underlying the poorer behavioural performance in children with a history of CM on neuropsychological tests were not optimally tapped by the tasks used. Third, the smaller group sizes for PM and MS may have resulted in less statistical power (e.g., leading to a lack of statistical difference between novel and familiar stimuli for these groups) and less reliable estimates of per cent impairments. Future studies using more advanced diagnostic techniques, a broader range of ERP tasks and larger group sizes can address these issues.

In conclusion, a significant proportion of children exposed to severe malaria have abnormal ERPs. The relationship between atypical ERPs and long-term educational and social outcomes need to be determined. Further studies to examine these deficits in detail and using neuro-imaging may be provide further insights into the brain damage caused by falciparum malaria and its link to atypical cognitive development.

## Figures and Tables

**Table 1:** Distribution of sample by diagnostic groups.

	Cerebral malaria (CM)	Malaria with seizures (MS)	Malaria with prostration (PM)	Unexposed children (CT)	*p*-value
Mean age (years);[SD (years)]	6.7 [0.5]	6.8 [0.5]	6.6 [0.6]	6.9 [0.5]	0.096
Attending school; yes (%)	9 (33%)	6 (43%)	3 (33%)	24 (31%)	0.655
Sex; male (%)	12 (44%)	7 (50%)	4 (44%)	38 (49%)	0.967
Maternal education; yes (%)	12 (44%)	9 (64%)	5 (56%)	38 (49%)	0.545

**Table 2:** Number of children in each diagnostic group with impaired performance on each ERP component in visual experiment.

	Diagnosis	*N*	Latency		Amplitude	
			Novels		Novels	
			Number impaired	Percentage	Number impaired	Percentage
P3a	CM	27	3	11	1	4
	CT	77	5	7	0	0
	MS	14	0	0	0	0
	PM	9	0	0	0	0
Nc	CM	27	Measures not available as Nc is an average computed over 350–850 ms		0	0
	CT	77			0	0
	MS	14			0	0
	PM	9			0	0

Children were impaired if their ERPs were outside of 2SD of mean group performance.

**Table 3:** Number of children in each diagnostic group with impaired performance on each ERP component in auditory experiment.

	Diagnosis	*N*	Latency		Amplitude	
			Novels		Novels	
			Number impaired	Percentage	Number impaired	Percentage
N2	CM	27	1	4	0	0
	CT	77	0	0	0	0
	MS	14	0	0	1	7
	PM	9	1	11	0	0
P3a	CM	27	0	0	1	4
	CT	77	3	4	2	3
	MS	14	0	0	2	14
	PM	9	0	0	1	11

Children are deemed cognitively impaired if their ERPs were outside of 2SD of mean group performance.

**Table 4:** Mean ERP peaks to the 3 types of pictures by diagnosis, averaged across midline (P3a, Nc) or temporal (N170) electrodes.

		Diagnosis	CM		MS		PM		CT	
			*N* = 27		*N* = 14		PM = 9		*N* = 77	
			Mean	SD	Mean	SD	Mean	SD	Mean	SD
Latency	N170	Novels	217	15.7	222	21.2	220	20.5	222	17.8
		Frequent	231	16.0	232	16.4	233	16.5	234	17.6
		Infrequent	228	19.7	229	16.7	227	15.5	230	19.0
	P3a	Novels	404	27.1	401	25.6	397	23.5	398	36.8
		Frequent	418	52.8	423	17.4	423	16.3	411	42.5
		Infrequent	423	14.7	422	19.6	418	11.0	416	38.3
Amplitude	N170	Novels	−5.18	3.9	−8.97	17.1	−1.76	4.0	−4.37	4.4
		Frequent	−7.83	5.2	−9.81	9.8	−8.44	4.8	−9.42	4.7
		Infrequent	−8.86	5.2	−6.01	9.6	−8.95	5.4	−10.39	6.5
	P3a	Novels	−1.40	10.6	−2.52	9.0	−4.70	7.3	2.39	6.9
		Frequent	3.76	7.6	−1.89	7.9	−0.34	4.7	4.74	6.3
		Infrequent	5.97	11.0	−0.48	11.4	0.51	6.1	7.99	8.4
	Nc	Novels	−3.07	7.4	−5.25	6.8	−5.19	5.0	−1.43	7.0
		Frequent	2.52	5.4	−0.19	6.7	−0.50	2.5	1.71	4.5
		Infrequent	4.65	9.0	2.53	10.1	4.06	8.6	4.45	6.8

CM represents cerebral malaria, CT represents unexposed, MS had malaria plus seizures and PM had malaria with prostration.

**Table 5:** Mean ERP peaks to the 3 types of sound by diagnosis, averaged across midline electrodes.

		Diagnosis	CM		MS		PM		CT	
			*N* = 27		*N* = 14		PM = 9		*N* = 77	
			Mean	SD	Mean	SD	Mean	SD	Mean	SD
Latency	P1	Novels	97	28.7	90	8.5	98	11.7	92	12.7
		Frequent	98	32.3	114	30.5	93	8.7	98	14.9
		Infrequent	91	10.0	98	14.5	96	16.2	93	15.0
	N2	Novels	226	34.8	239	18.4	248	21.5	238	18.3
		Frequent	226	30.4	228	32.7	240	11.0	239	15.9
		Infrequent	230	13.7	237	19.2	234	17.3	230	18.9
	P3a	Novels	313	20.7	327	20.6	326	13.2	319	24.3
		Frequent	308	18.2	313	13.7	316	20.6	319	25.9
		Infrequent	311	17.7	315	21.5	320	25.5	314	24.1
Amplitude	P1	Novels	6.96	5.3	7.85	5.5	8.04	4.6	7.72	4.7
		Frequent	8.27	5.5	4.54	6.5	10.49	3.9	7.28	4.3
		Infrequent	6.22	5.7	8.16	4.7	8.21	4.6	6.98	4.0
	N2	Novels	−1.92	6.1	0.16	8.7	2.66	4.6	−0.06	7.1
		Frequent	−10.07	6.8	−7.98	7.7	−5.12	3.6	−8.42	4.6
		Infrequent	−11.68	5.6	−7.25	4.6	−7.23	4.3	−7.81	5.6
	P3a	Novels	3.52	6.3	2.65	9.0	5.14	7.0	8.65	6.9
		Frequent	−2.02	6.0	−2.73	5.2	0.32	4.0	−0.87	4.7
		Infrequent	−1.63	4.5	−0.70	8.4	0.00	3.7	−0.35	5.0

CM represents cerebral malaria, CT represents unexposed, MS had malaria plus seizures and PM had malaria with prostration.

**Table 6:**
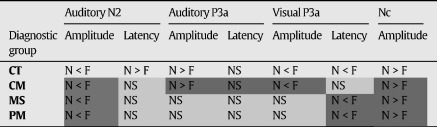
Response to novel compared to familiar stimuli for auditory N2 and P3a and visual P3a and Nc by diagnosis.

N = Novel, F = Infrequent Familiar or Frequent Familiar, NS = Not significant; Dark grey cells reflect a pattern similar to control group, and light grey cells reflect an atypical response.
